# Laparoscopic duodenum–preserving pancreatic head resection with real-time indocyanine green guidance of different dosage and timing: enhanced safety with visualized biliary duct and its long-term metabolic morbidity

**DOI:** 10.1007/s00423-022-02570-0

**Published:** 2022-07-19

**Authors:** Chao Lu, Biwu Xu, Yiping Mou, Yucheng Zhou, Weiwei Jin, Tao Xia, Yuanyu Wang, Qicong Zhu, Zhiqin Fu

**Affiliations:** 1 Department of Clinical Medicine, Medical College of Soochow University, Suzhou, 215006 Jiangsu People’s Republic of China; 2 Department of General Surgery, Cancer Center, Division of Gastrointestinal and Pancreatic Surgery, Zhejiang Provincial People’s Hospital, Affiliated People’s Hospital, Hangzhou Medical College, Hangzhou, 310014 Zhejiang China; 3 Key Laboratory of Gastroenterology of Zhejiang Province, Hangzhou, 310014 Zhejiang China; 4 Bengbu Medical College, Bengbu, 233030 Anhui China; 5 The Cancer Hospital of the University of Chinese Academy of Sciences (Zhejiang Cancer Hospital), Institute of Basic Medicine and Cancer (IBMC), Chinese Academy of Sciences, Hangzhou, 310022 Zhejiang China

**Keywords:** Laparoscopic, Pancreatic head, Fluorescence imaging, Pancreatic dysfunction

## Abstract

**Purpose:**

Laparoscopic duodenum–preserving pancreatic head resection (L-DPPHR) is technically demanding with extreme difficulty in biliary preservation. Only a few reports of L-DPPHR are available with alarming bile leakage, and none of them revealed the long-term metabolic outcomes. For the first time, our study explored the different dosage and timing of indocyanine green (ICG) for guiding L-DPPHR and described the long-term metabolic results.

**Methods:**

Between October 2015 and January 2021, different dosage and timing of ICG were administrated preoperatively and evaluated intra-operatively using Image J software to calculate the relative fluorescence intensity ratio of signal-to-noise contrast between bile duct and pancreas. Short-term complications and long-term metabolic disorder were collected in a prospectively maintained database and analyzed retrospectively.

**Results:**

Twenty-five patients were enrolled without conversion to laparotomy or pancreaticoduodenectomy. Administrating a dosage of 0.5 mg/kg 24 h before the operation had the highest relative fluorescence intensity ratio of 19.3, and it proved to guide the biliary tract the best. Fifty-six percent of patients suffered from postoperative complications with 48% experiencing pancreatic fistula and 4% having bile leakage. No one suffered from the duodenal necrosis, and there was no mortality. When compared with the non-ICG group, the ICG group had a comparable diameter of tumor and similar safety distance from lesions to common bile duct; however, it decreased the incidence of bile leakage from 10% to none. The median length of hospital stay was 16 days. After a median follow-up of 26.6 months, no one had tumor recurrence or refractory cholangitis. No postoperative new onset of diabetes mellitus (pNODM) was observed, while pancreatic exocrine insufficiency (pPEI) and non-alcoholic fatty liver disease (NAFLD) were seen in 4% of patients 12 months after the L-DPPHR.

**Conclusion:**

L-DPPHR is feasible and safe in selected patients, and real-time ICG imaging with proper dosage and timing may greatly facilitate the identification and the prevention of biliary injury. And it seemed to be oncological equivalent to PD with preservation of metabolic function without refractory cholangitis.

## Introduction

Pancreaticoduodenectomy (PD) remains the standard management for pancreatic head tumors. With the development of advanced laparoscopic skills, laparoscopic pancreaticoduodenectomy (L-PD) prevails in experienced hands with minimal invasive advantages [1]. However, L-PD was considered too aggressive to destroy the integrity of the duodenum and bile duct “innocently” for non-invasive tumors. Moreover, it complicated with alarming long-term metabolic dysfunction [2, 3] and high rate of refractory biliary complications (18.9%) [4, 5], especially for patients with longer life expectancy and higher demands for quality of life.

Duodenum-preserving pancreatic head resection (DPPHR) was first introduced by Beger in 1972 [6]. Attempts to perform Laparoscopic DPPHR(L-DPPHR) was made amidst the soaring passion for laparoscopic pancreatic surgery [7–13]. However, limited studies indicated alarming biliary leaks which varied from 11.8 to 16.7% [8–13], greatly restricting its applications because of the fear of injuring the invisible bile ducts embedded in the pancreas. In 2009, Ishizawa introduced the usage of indocyanine green (ICG) fluorescence imaging to delineate the bile duct intra-operatively, and it enabled real-time identification of biliary anatomy to avoid biliary injury [14]. Inspired by Ishizawa and Cai [9], ICG can be adopted to identify the invisible common bile duct in real-time. However, few studies reported the detailed techniques for ensuring the integrity of the bile duct. Moreover, no study reported the long-term metabolic morbidity of L-DPPHR, which only proved “organ preserving” rather than “function preserving.”

To the best of our knowledge, this is the first study to report the metabolic morbidity after L-DPPHR, which is also the first and foremost outcome concerned by both surgeons and patients. And it may raise the awareness and fill in the blanks of the long-term outcomes of this promising alternative. Moreover, prior studies rarely proposed the objective method to evaluate the efficacy of fluorescent cholangiography with different dosage and timing of ICG, and they hardly offered the individualized dosage and timing when using ICG, which may be of great value to visualize and preserve the bile duct with more obvious contrast to identify. In this study, we also tried to explore proper dosage and timing of ICG use, hoping to solve the “Achilles heel” which restrains its routine practice.

## Materials and methods

### Patients and methods

Patients diagnosed with benign or low-grade malignant lesions were indicated for L-DPPHR. All the patients who underwent L-DPPHR between October 2015 and January 2021 in Zhejiang Provincial People’s Hospital were enrolled. And the specific indications were listed below: (1) pancreatic cystic lesions with recurrent pancreatitis; (2) cystic size larger than 3 cm with thickened and enhanced cystic wall; (3) endoscopic ultrasound confirmed solid component or mural nodule; (4) dilated main pancreatic duct; (5) cystic growth with elevated CA19-9 in serum; and (6) suspected solid pseudo-papillary tumor with any size or neuroendocrine tumors ≤ 2 cm without radiological evidence of lymph node metastasis or distant metastasis. Computed tomography (CT) and magnetic resonance cholangiopancreatography (MRCP) were routinely evaluated together with endoscopic ultrasound (EUS) outcomes. All the clinical tests and images were evaluated by multi-disciplinary team (MDT) to confirm the indications for surgery. Moreover, the safety distance was defined by the minimal distance between lesions and bile ducts calculated by two separate radiologists. Endoscopic naso-biliary drainage (ENBD) was selectively placed. And the indications for ENBD included: 1. Diameter of lesions in pancreatic head was larger than 5 cm; 2. Safety distance between lesions and bile ducts was less than 3 mm. This study was approved by the Institutional Review Board and Ethics Committees of Zhejiang Provincial People’s Hospital. And all the patients were informed with the written consents in accordance with the Declaration of Helsinki.

All the clinical data was retrospectively analyzed using a prospectively maintained database, including baseline characteristics, 90-day morbidity, mortality, and long-term metabolic dysfunctions. All the patients were divided into ICG guidance group and non-ICG group, and all the clinical outcomes were analyzed. Postoperative pancreatic fistula (POPF) and postoperative hemorrhage (POPH) were defined and classified by the International Study Group in Pancreatic Surgery (ISGPS) criteria [15]. Bile leakage was defined and classified by the International Study Group of Liver Surgery (ISGLS) criteria [16]. Moreover, Clavien-Dindo classification was adopted to grade the complications [17]. Refractory cholangitis was defined as recurrent systemic inflammation which had fever or jaundice with abnormal liver function and biliary imaging evidence [4].

### Different dosage and timing for ICG

Indocyanine green was administrated preoperatively by intravenous injections and intra-operatively by intra-biliary injections through ENBD. For intravenous way, different dosage ranging from 1, 0.5 to 0.25 mg/kg were adopted. Moreover, different timing with 12 h, 24 h, or 36 h was used before surgery. However, for the patients with high risk of bile duct injury, the intra-biliary way was used with fixed doses of 12.5 mg/5 ml injected by ENBD to detect the suspicious site of injury.

### Assessment of common bile duct detectability and efficacy using fluorescent cholangiography

**Fig. 1:**
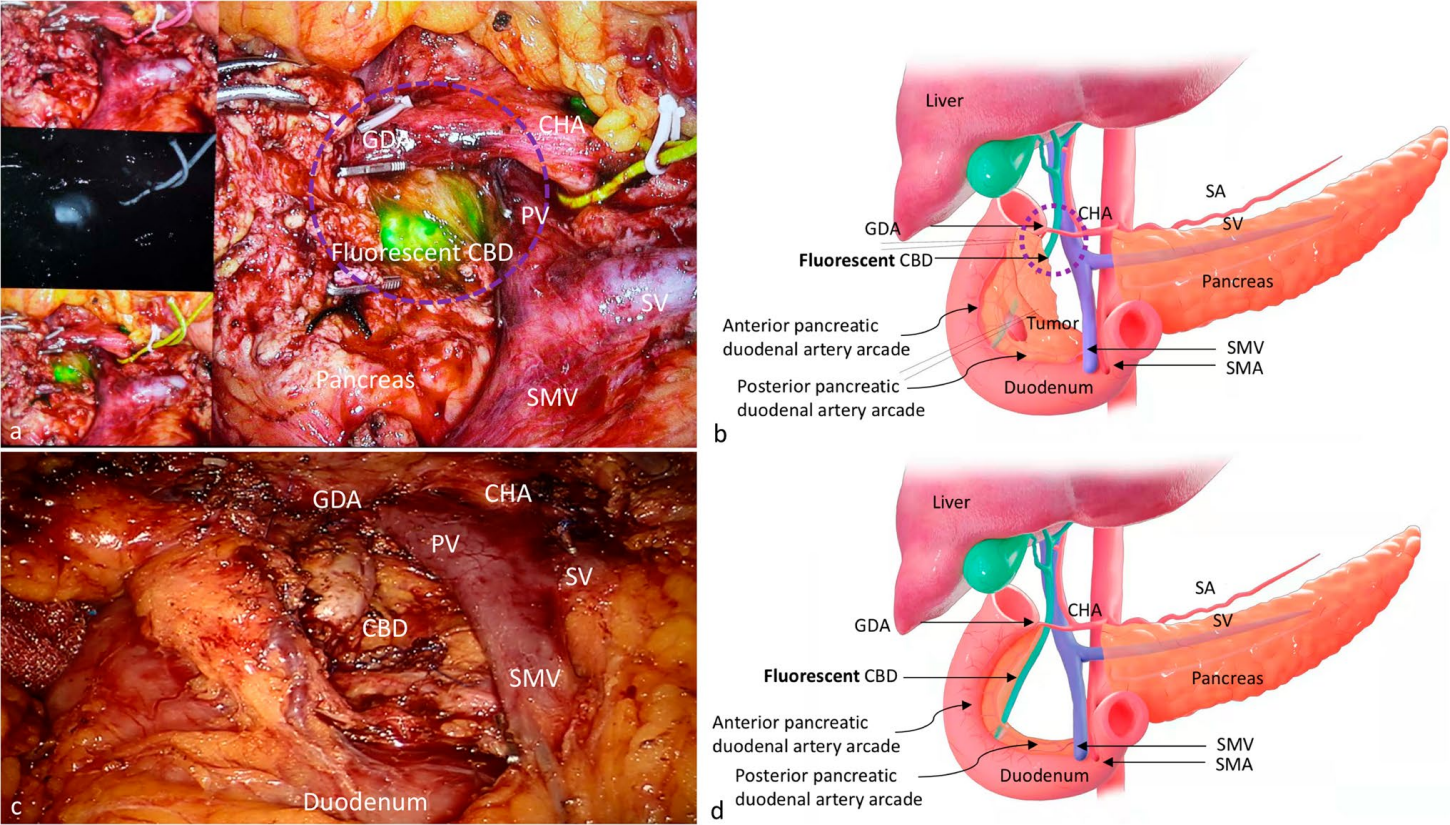
Real-time indocyanine green–guided laparoscopic duodenum-preserving pancreatic head resection. (**a**) The crucial plane of using fluorescent cholangiography to detect the common bile duct. The circle represents the targeted area to identify the fluorescent CBD. (**b**) The schema to illustrate the crucial plane of using fluorescent cholangiography. (**c**) Completion of pancreatic head resection with preserving the duodenum, common bile duct and pancreatic duodenal artery arcades. (**d**) The schema to illustrate the completion of pancreatic head resection CHA, common hepatic artery; GDA, gastroduodenal artery; SA, splenic artery; SMA, superior mesenteric artery; PV, portal vein; SMV, superior mesenteric vein; SV, splenic vein; CBD common bile duct

The fluorescent cholangiography was routinely performed after the transection of pancreatic neck. The detectability of common bile duct (CBD) was assessed by the identification of CBD by fluorescent imaging among the targeted area surrounded by common hepatic artery, gastroduodenal artery, and the right side of the superior mesenteric vein-portal vein (Fig. 1a, b). The accuracy of detection of the common bile duct was evaluated by two surgeons before and after the resection of the pancreatic tumor. Whether the “suspected” bile duct before the resection was consistent with the “confirmed” bile duct or not was evaluated.

As for the assessment of efficacy for fluorescent cholangiography with different dosage and timing of ICG, we calculated the relative fluorescence intensity ratio of signal-to-noise contrast between the bile duct and pancreas by the open-source Image J 64 quantitative software (National Institutes of Health, Bethesda, MD) in the monochromatic fluorescence mode. We calculated absolute values of 5 random points both in the common bile duct and pancreas for each, and the fluorescence intensity ratio was achieved by the ratio of mean values between the common bile duct and pancreas.

### Surgical techniques

The patient was placed in a reversed Trendelenburg position. Carbon dioxide pneumo-peritoneum was established (15 mmHg) using a Veress needle. We routinely used the modified placement of five trocars as reported [18], with one observing trocar inferior to umbilicus and the other four trocars symmetrically distributed at the mid-clavicular line and anterior axillary line.

After exploration of the whole abdomen, the gastro-colic ligament was divided first, and then the Kocher maneuver was made but incompletely to protect the posterior superior pancreaticoduodenal artery (PSPDA). After that, we mobilized the superior border and the inferior border of pancreas to expose the common hepatic artery (CHA) and the superior mesenteric vein-portal vein (SMV-PV), respectively. Along the CHA and gastroduodenal artery (GDA), the anterior superior pancreaticoduodenal artery (ASPDA) was separated and preserved, only branches to the pancreatic head were ligated. At the same time, the retro-pancreas tunnel was created.

Then the distal end of pancreas was transected. Of particular note, the main pancreatic duct (MPD) distal to the lesion should be identified and cut with scissor rather than the ultrasound scalpel to avoid stenosis. Before the resection of the tumor, an intra-operative laparoscopic ultrasound was performed to locate the lesion and set up the planed resection margin. Afterwards, real-time ICG fluorescence imaging was used to visualize the common bile duct and guide the resection of pancreatic parenchyma while preserving the CBD intact. Of great caution, the uncinate and pancreatic head was transected gradually along the duodenum with the preservation of the branches of both anterior and posterior inferior pancreaticoduodenal artery (IPDA), especially the blood supply to the duodenum (Fig. 1c, d). Then the proximal end of the MPD was identified and ligated nearby the ampulla. Then the specimen and lymph node sampling were retrieved, and the intra-operative frozen section was sent to confirm the diagnosis with negative margin and exclusion of malignancy. After the removal of the specimen, fluorescent cholangiography was also performed to detect the suspicious injury of the bile duct. Finally, a direct pancreaticogastrostomy or Roux-en-Y end-to-side pancreaticojejunostomy (duct to mucosa) was performed [7]. Two drainage tubes were placed behind the anastomosis.

### Metabolic dysfunction and follow-up

The metabolic dysfunction was separated into endocrine dysfunction and exocrine dysfunction. Endocrine comorbidity was evaluated by the postoperative new onset of diabetes mellitus (pNODM), defined by the presence of abnormal blood glucose postoperatively with history of normal preoperative fasting blood glucose (FBG). More specifically, elevated FBG and oral glucose tolerance test (OGTT) were measured. On the other hand, the exocrine morbidity was named postoperative pancreatic exocrine insufficiency (pPEI), defined by the symptoms of steatorrhea and weight loss with normal diet intake and relieved by the supplement of pancreatic enzyme tablets. Non-alcoholic fatty liver disease (NAFLD) was also used to evaluate the pPEI, defined by absolutely decreased CT value of less than 40 Hounsfield units (HU) or relatively declined CT values of at least 10 HU lower than that of the spleen. Regular follow-ups were carried out every 3 months by medical records and phone calls. And CT scans were evaluated every 6 months.

## Results

### Demographic and clinico-pathological characteristics

Twenty-five patients who underwent L-DPPHR were enrolled between October 2015 and January 2021. All the demographic characteristics are listed in Table 1. Among them, there were 8 male patients and 17 female patients. The average age was 46.7 ± 17.7 year-old with a median body mass index of 21.9 kg/m^2^. All the lesions were located in the pancreatic head, and the average diameter of the tumor was 3.7 ± 1.7 cm with the average safety distance from the lesions to CBD 6.5 ± 3.1 mm. The preoperative liver functions were all normal. Pathological outcomes confirmed 7 solid pseudo-papillary neoplasms (SPN), 8 intraductal papillary mucinous neoplasms (IPMN), 5 serous cystic neoplasms (SCN), and 5 pancreatic neuroendocrine tumors (pNET). The details of the 5 patients diagnosed with pNET are presented in Table 2. Compared with the non-ICG group, all the demographic data showed no significant difference. To be specific, these two groups had comparable age, similar diameter of tumor mass, and comparable distance from the lesions to the common bile duct.

**Table 1:** Demographic characteristics of patients underwent L-DPPHR with or without ICG guidance

Variables	Total	ICG group	Non-ICG group	*P* value
No. of patients	25	15	10	/
Male/female	8/17	4/11	4/6	0.67
Age (year-old)	46.7 ± 17.7	43.2 ± 20.2	51.9 ± 12.5	0.24
BMI (Kg/m^2^, median)	21.9	23.0	21.3	0.28
Tumor diameter (cm)	3.7 ± 1.7	3.7 ± 2.0	3.8 ± 1.4	0.84
Distance from lesions to CBD (mm)	6.5 ± 3.1	6.5 ± 3.5	6.4 ± 2.4	0.92
Main pancreatic duct diameter (mm)	2.1 ± 1.1	2.1 ± 1.1	2.1 ± 1.1	0.87
Pathological diagnosis				
SPN	7	5	2	0.30
IPMN	8	4	4	
SCN	5	1	4	
pNET	5	5	0	

*BMI* body mass index, *CBD* common bile duct, *SPN* pseudo-papillary neoplasms, *IPMN* intraductal papillary mucinous neoplasms, *SCN* serous cystic neoplasms, *pNET* pancreatic neuroendocrine tumors

**Table 2:** Detailed tumor characteristics and margin status of pancreatic neuroendocrine tumors (pNET)

	Histology	Size (cm)	Grade	Ki-67(%)	Mitosis	Margin status	Lymph node
1	Non-functional	2	G1	< 1%	< 1/10 HPF	Negative	0/2
2	Insulinoma	1.2	G1	1%	< 2/10 HPF	Negative	0/0
3	Insulinoma	1.6	G2	3%	< 2/10 HPF	Negative	0/0
4	Non-functional	1.0	G1	1%	< 1/10 HPF	Negative	0/2
5	Non-functional	1.8	G1	2%	< 2/10 HPF	Negative	0/2

### Detection of common bile duct using fluorescent cholangiography and its efficacy

**Fig. 2:**
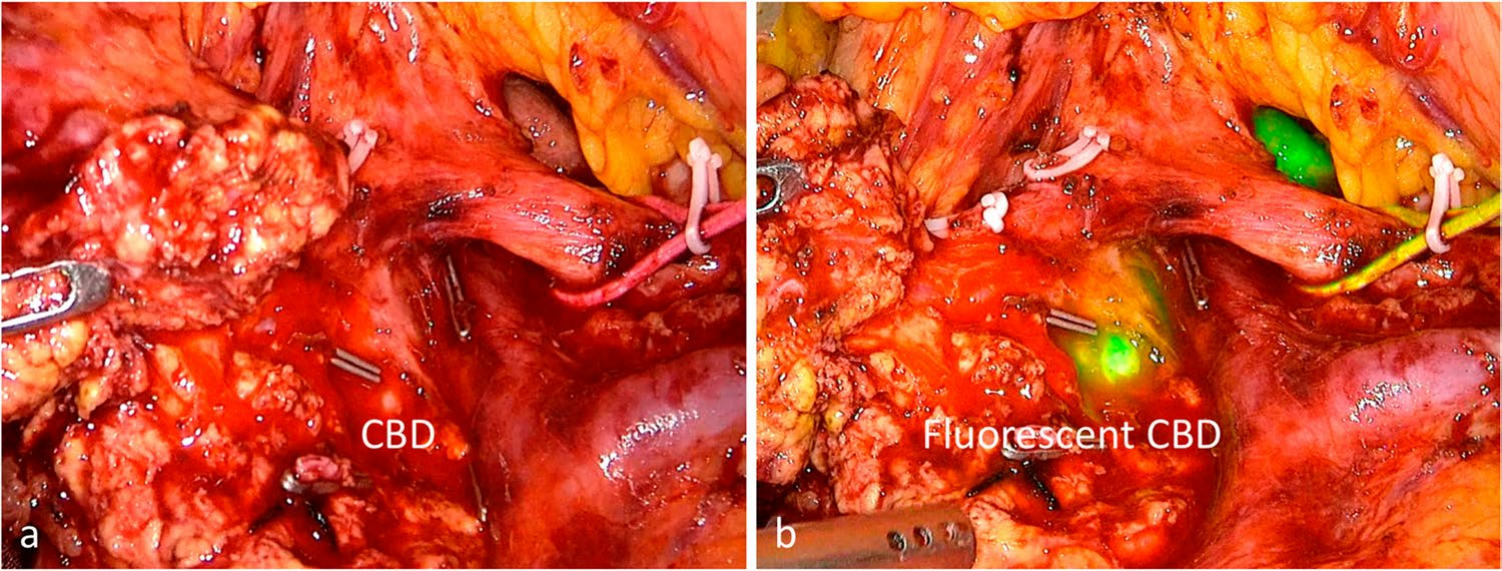
Contrast of identifying the common bile duct (CBD) using ICG fluorescent cholangiography or not (**a**) identify the CBD without the help of ICG fluorescent cholangiography; (**b**) identify the CBD using the ICG fluorescent cholangiography

Among all the 25 patients, 15 of them received real-time ICG-guided surgery, while the rest of the 10 were not. Among the ICG-guided group, fluorescent cholangiography successfully identified 14 (93.3%) CBD in the targeted area with totally 100% accuracy between “Suspicious” CBD and “Confirmed” CBD. Only 1 (6.7%) patient had a severe background noise of “whole green” view with difficulty in distinguishing the CBD from the pancreas. While, for the rest of the 10 patients without fluorescent guidance, only 5 (50%) patients actually identified CBD in the targeted area without fluorescent cholangiography, and 3 of the 5 achieved consistence between “Suspicious” CBD and “Confirmed” CBD (Fig. 2). ICG guidance increased both the ability of detection (93.3% vs. 50%, *P* = 0.045) and the accuracy of identification (100% vs. 60%, *P* = 0.098) using fluorescent cholangiography than that of naked observation without ICG.

**Fig. 3:**
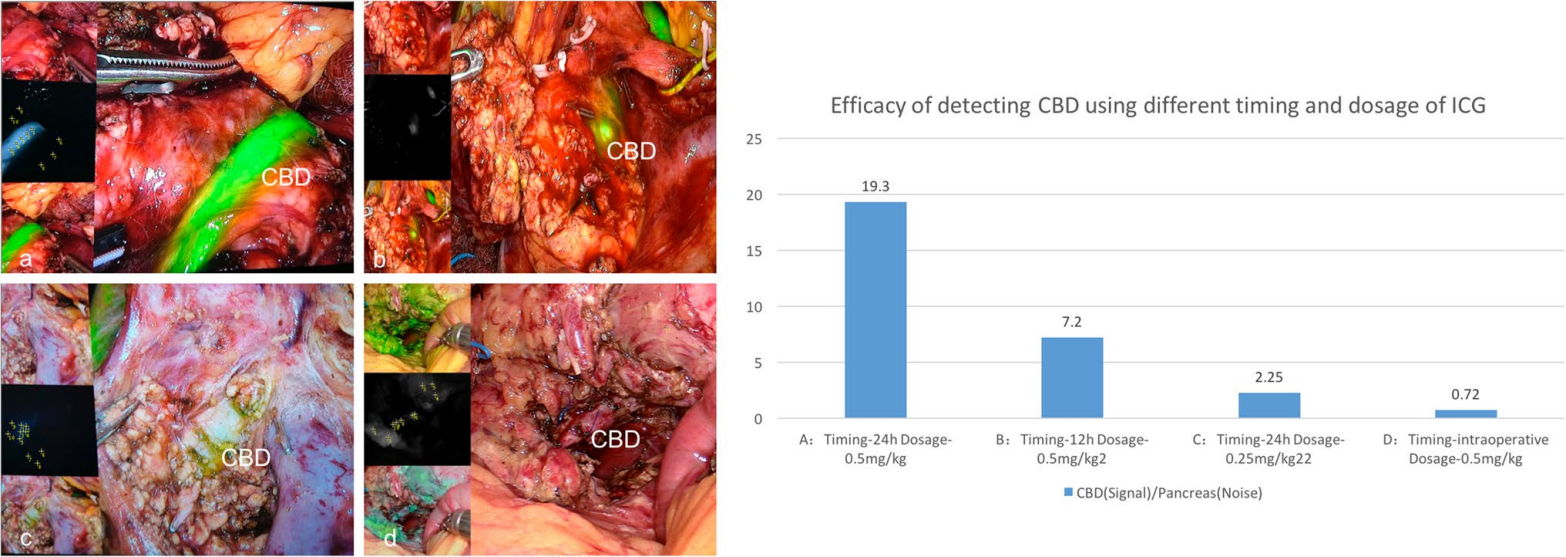
Different timing and dosage of indocyanine green and its efficacy evaluated by the relative fluorescence intensity ratio. Take figure (a) for example, Image J software was used to calculate the intensity of common bile duct (points 1–5, 150.2 in average) and the intensity of pancreas (points 6–10, 7.8 in average), and the fluorescence intensity ratio was 150.2/7.8 = 19.3. (**a**) Timing: injected intravenously 24 h before the operation; dosage: 0.5 mg/kg; (**b**) timing: injected intravenously 12 h before the operation; dosage: 0.5 mg/kg; (**c**) timing: injected intravenously 24 h before the operation; dosage: 0.25 mg/kg; (**d**) timing: injected intravenously at the beginning of the operation (intraoperative); dosage: 0.5 mg/kg

As for the efficacy of ICG application with different timing and dosage, relative fluorescence intensity ratio was calculated. The application of ICG with timing of 24 h preoperatively and dosage of 0.5 mg/kg had the highest signal-to-noise ratio (19.3), followed by the other timing and dosage (Fig. 3). Moreover, it was interesting to find that intra-operative use of the ICG with a dosage of 0.5 mg/kg produced a severe noise with relative fluorescence intensity ratio of only 0.72; this proved that improper use of the ICG may enhance the noise and cause chaos and create difficulty to identify the CBD in return.

### Operative outcomes and complications

All the 25 patients underwent L-DPPHR without conversion to laparotomy or L-PD (Table 3). Only 1 patient underwent total L-DPPHR, with the rest of the twenty-four patients receiving partial L-DPPHR with preservation of parenchyma along the SPDA-IPDA arcades. Five patients underwent pancreaticogastrostomy, and the rest of the 20 patients received pancreaticojejunostomy. The average operative time was 329.2 ± 30.8 min, and the mean blood loss was 101.2 ± 47.7 ml. No patient required transfusion. The mean out-of-bed, first-anus-exhaust, and first liquid intake occurred 1.6 ± 0.6 days, 2.8 ± 0.7 days, and 4.1 ± 2.0 days, respectively.

**Table 3:** Operative outcomes and short-term postoperative outcomes of patients underwent L-DPPHR with or without ICG guidance

Variables	Total	ICG group	Non-ICG group	*P* value
Operative times (min)	329.2 ± 30.8	324 ± 26.6	337 ± 36.4	0.62
EBL (ml)	101.2 ± 47.7	88 ± 45.7	121 ± 45.8	0.12
Conversion to laparotomy or PD	0	0	0	1.00
Surgical types				
Total L-DPPHR	1	1	0	0.86
Partial L-DPPHR	24	14	10	
Anastomosis types				
Pancreaticogastrostomy	5	5	0	0.06
Pancreaticojejunostomy	20	10	10	
Complications	56% (14)	53.3% (8)	60% (6)	0.93
Pancreatic fistula	48% (12)	53.3% (8)	40% (4)	0.61
Biochemical leak	24% (6)	26.7% (4)	20% (2)	
Grade B	20% (5)	20% (3)	20% (2)	
Grade C	4% (1)	6.6% (1)	0%	
Bile leakage	4% (1)	0%	10% (1)	0.40
Clavien-Dindo classification				
I–II	48% (12)	46.7% (7)	50% (5)	0.92
III	8% (2)	6.6% (1)	10% (1)	
IIIa	4% (1)	0%	10% (1)	
IIIb	4% (1)	6.6% (1)	0%	
IV–V	0%	0%	0%	
Reoperation	4% (1)	6.6% (1)	0%	0.40
30-Day mortality	0%	0%	0%	1.00
Median length of hospital stay	16 (8–87)	16 (8–42)	16.5 (9–87)	0.96

*EBL* estimated blood loss, *PD* pancreaticoduodenectomy, *L-DPPHR* laparoscopic duodenum–pre-serving pancreatic head resection, *ICG* indocyanine green

There were 56% (14) of patients suffering from postoperative complications. Pancreatic fistula occurred in 48% (12) of patients, including 24% (6) biochemical leak POPF, 20% (5) grade B POPF, and 4% (1) grade C POPF. Among all the grade B POPF, one combined with POPH and received digital subtraction angiography (DSA); the other four patients had extended drain placement longer than 3 weeks without extra puncture. Bile leakage occurred in one (4%) patient. Of note, this patient did not use the ICG and had difficulty in identifying the CBD. No one suffered from the duodenal necrosis and no perioperative deaths occurred. The median length of hospital stay was 16 days (range 8–87 days).

To be detailed, 2 patients experienced severe complications (Clavien-Dindo Grade III). One suffered from both Grade B POPF and Grade B bile leakage resulting in pseudo-aneurysm in GDA and finally controlled by DSA and embolization without re-operation on postoperative day (POD) 27, and with efficient drainage, he was discharged uneventfully on POD 87. The other one experienced Grade C POPF from pancreaticogastrostomy leakage and required reoperation on POD10 with the removal of the remnant pancreas while preserving the spleen, and finally discharged on POD32.

The use of ICG did not prolong the operative time nor increased the complications. On the contrary, the ICG group had lower incidence of bile leakage than that of the non-ICG group (10% vs. 0%, *P* = 0.4). Although the difference failed to reach a significant difference, the decrease of incidence from 10% to none was sure to have clinical value. As to the anastomosis style, 40% (2/5) of patients are complicated with pancreatic fistula in the pancreaticogastrostomy group, and 50% (10/20) suffered from it in the pancreaticojejunotomy group. When compared, there was no significant difference between pancreaticojejunotomy group and pancreaticogastrostomy group (*P* = 0.9).

### Follow-up and long-term metabolic dysfunctions (pNODM /pPEI/NAFLD)

The median follow-up was 26.6 months (ranging from 8.4 to 77.7 months) without loss. None suffered from recurrence or metastasis. During the follow-up, all the patients were free of refractory cholangitis and dilation of CBD. Moreover, in terms of endocrine dysfunction, no pNODM was observed in the long-term follow-up. However, in the view of exocrine insufficiency, pPEI and NAFLD were complicated in 4% (1) patient 12 months after the L-DPPHR (Table 4), with the supplement of pancreatic enzyme, steatorrhea and weight loss relieved, but NAFLD was not.

**Table 4:** Long-term outcomes and metabolic morbidity of L-DPPHR

Variables	Total
Median follow-up (months)	26.6 (8.4 to 77.7)
Tumor recurrence	0%
Functional variables	
Refractory cholangitis	0%
Pancreatic metabolic dysfunction	4%
Endocrine dysfunction (pNODM)	0%
Exocrine dysfunction (pPEI/NAFLD)	4%

*pNODM* postoperative new onset of diabetes mellitus, *pPEI* pancreatic exocrine insufficiency, *NAFLD* non-alcoholic fatty liver disease

## Discussion

DPPHR was initially introduced for chronic pancreatitis by Beger [6] and was proven to decrease the long-term endocrine and exocrine dysfunction compared with pancreaticoduodenectomy (PD) [2, 3]. However, in terms of laparoscopic DPPHR, our team reported the first case in 2016 [7]; later on, a few studies reported 12–24 cases are complicated with astonishing biliary leakages of 4.5–16.7% without exception [8–10]. Moreover, none of them reported the long-term metabolic dysfunction, which was of great importance on deciding whether to perform L-DPPHR or not. To identify the bile duct, Cai et al. firstly reported the innovative ICG-guided L-DPPHR [9, 19]. However, we tried and found that the suggested dosage and timing of ICG caused severe background noise and impaired the identification of the bile duct from the pancreas in return, which was awaited to be modified.

To the best of our knowledge, this study reported the largest number of L-DPPHR to date. Moreover, this study firstly reported the long-term metabolic morbidity of DPPHR in the laparoscopic setting, providing more evidence on not only “organ preserving,” but also “function preserving” as well. What is more, we explored and proposed the proper dosage and timing of using ICG to visualize and preserve the bile duct more certainly, which helped to decrease the incidence of biliary leakage and improve the outcomes.

In 2018, Cao et al. reported the first successful series of L-DPPHR; however, the bile leakage occurred in 16.7% of the patients [8]. The same was also true for robotic DPPHR, Peng et al. observed 11.8% of the patients complicated with biliary leak [12]. Beger also demonstrated 8.4% biliary complications in open DPPHR [20]. The invisible bile duct embedded in the pancreas makes the CBD extremely hard to identify during the operation. Once injured, biliary leakage may result in lethal danger combined with the higher risk of pancreatic fistula. Cai et al. firstly introduced ICG into real-time imaging of CBD during L-DPPHR [9], but the bile leakage remained high in 12.5% of the patients. We followed the timing of ICG they suggested at the beginning of the operation, but we found that severe background green existed and impaired the identification of the CBD. Just as the intra-operative use of ICG in our study, it had a relative fluorescence intensity ratio 0.72; this proved that improper use of the ICG may enhance the noise and create extra difficulty to identify the CBD in return. Such confusion inspired us to modify the dosage and timing of ICG to enhance the ability to identify the CBD more obviously using fluorescence cholangiography.

However, no study evaluated the efficacy of fluorescent cholangiography with different timing and dosage of ICG before, and there was no suitable method to evaluate the efficacy objectively. Firstly, in our study, we innovatively adopted the open-source Image J 64 quantitative software to calculated signal-to-noise contrast between bile duct and pancreas, which made the assessment more objective. Based on the relative fluorescence intensity ratio under different dosage and timing of ICG, we concluded that the ICG dosage of 0.5 mg/kg with timing of 24 h before the surgery had the most obvious signal-to-noise (CBD-to-pancreas) contrast with the highest fluorescence intensity ratio of 19.3 in fluorescent cholangiography, and this strategy of ICG use guided the surgery more efficiently to detect the bile duct.

ICG guidance with the help of fluorescent cholangiography enhanced the detectability and accuracy to identify the bile duct. As a result, ICG guidance decreased the biliary leakage. Compared with the non-ICG group, diameters of tumor mass of ICG group were similar and the distances from the lesions to CBD were comparable, from which we may infer that the difficulty to operate L-DPPHR and to identify the common bile duct were alike. However, ICG guidance increased both the ability of detection (93.3% vs. 50%, *P* = 0.045) and the accuracy of identification (100% vs. 60%, *P* = 0.098) using fluorescent cholangiography than that of naked observation. And the obvious contrast of CBD and pancreas using ICG guidance surely elevated the detectability and promoted the safety in biliary preservation (Fig. 2). Although the decrease of biliary leakage rate from 10% to none failed to reach a significant difference, it should be interpreted into invalidity of the sample size. What is more, the only patient suffering from the bile leakage in our study encountered difficulty in identifying the CBD intra-operatively and finally resulted in biliary injury, which might be avoidable with the help of ICG guidance. In general, ICG fluorescent cholangiography surely enhanced the safety of L-DPPHR in terms of the biliary complication with visualized biliary duct.

However, unlike other studies reported in the literatures, the short-term complication reached to 56%, and it was slightly higher than that of our routine L-PDs. But the major complications (Clavien-Dindo III–V) were comparable with an incidence of 8%. Previously in our 320 consecutive L-PDs, only 32.2% of the patients developed morbidity with 10.9% being major complications [21]. This may be attributed to the higher occurrence of pancreatic fistula in 48% of the patients, and the possible explanation may be the soft tissue of the remnant pancreas and un-dilated pancreatic duct with mean diameter of 2.1 mm together with an extended surface of pancreatic parenchyma along the duodenum and CBD. Similarly, Cai et al. reported elevated POPF in 45.8% of the L-DPPHR patients with only 4.2% Grade B POPF [9]. Others observed an overall complication of 20–36.4% with 6.7–16.7% POPF after L-DPPHR [8, 10, 13, 20]. As high as the POPF rate was, only 8% (2) of the patients required intervention, including one required reoperation and the other one controlled by DSA. However, the rest 40% (10) of patients recovered without repositioned drainage by percutaneous puncture. Usually, we closed the cutting surface carefully for the preserved pancreas along the duodenum and behind the CBD with interrupted suture. Generally, L-DPPHR led to higher incidence of POPF, but it was controllable and acceptable.

In terms of the metabolic function, our study indicated that L-DPPHR held the advantages of maintenance of both the endocrine and exocrine functions. From the aspect of endocrine dysfunction, it was reported in the literatures that 10.6–15.7% of patients developed pNODM after PD for benign tumors [2, 3, 22]. However, only 5–6% of the patients developed pNODM after DPPHR [2, 3]. Although the incidence of pNODM after L-PD remained unreported, our data demonstrated no pNODM after L-DPPHR, and it was extremely inspiring for the preservation of the endocrine function. The same was also true for the exocrine dysfunction, our data revealed that L-DPPHR complicated with only 4% pPEI and NAFLD, and it was much lower than that of PD with pPEI in 44.9–50.4% and NAFLD in 23.7% of the patients [2, 3, 22]. However, the incidence of pPEI and NAFLD after DPPHR was 6–6.7% [2, 3] and 3.03% [2], respectively, which was comparable with that of our data on L-DPPHR. Beger presumed that it was sparing the duodenum rather than the pancreas that caused metabolic dysfunction, because the resection of pancreatic parenchyma was comparable for PD and DPPHR [2, 3]. He believed that the preservation of the duodenopancreatic neural connections ensured an unchanged release of endo/exocrine regulating hormones, which maintained the function as well. Moreover, our study indicated that L-DPPHR seemed to have lower metabolic dysfunction than that of open DPPHR, which may attribute to the minimal invasive advantages of magnification with a better view of anatomy, and more precise dissection as well. However, such conclusions should be drawn carefully by more convincing research later.

Refractory cholangitis was also far from rare in long-term follow-up, and it exerted a great negative impact on the quality of life, especially for patients with longer life expectancy unlimited by the tumor malignancy. Hiroki et al. observed an incidence of 18.9% cholangitis mostly within 1 year after PD, and half of them developed biliary strictures [4]. Moreover, such patients usually had un-dilated bile duct, which exerted great risks on the early complications of the bile leakage. Pauline et al. found that a thin bile duct (< 5 mm) was the predominantly risk factor for developing the bile leakage (3.3%) and stricture (4.3%) [4]. In our study, the average diameter of CBD was only 6.8 ± 1.2 mm, and it was of great difficulty for surgeons to perform biliary-enteric anastomosis. Furthermore, the CBD was usually resected “innocently” even taking the oncological outcomes into consideration. L-DPPHR preserves not only the CBD intact but the Oddi’s sphincter as well. Unsurprisingly, no refractory cholangitis was observed during the long-term follow-up.

Last but not least, unlike the management of chronic pancreatitis, the oncological outcomes outweigh its short-term outcomes and dysfunction morbidity for such patients. The indications of L-DPPHR for pancreatic neuroendocrine tumors (pNET) were controversial and strictly selected in our center. pNET exhibit heterogeneous biologic behavior from indolent to aggressive [23], so we routinely resected the pancreatic head instead of enucleation for certainly negative resection margins. Moreover, intra-operative lymph node sampling around the lesions and frozen section were routinely performed to rule out malignancy. However, the exact method for lymph node sampling needs further investigation.

In particular, all the patients were free of recurrence in the long-term follow-up. So we concluded that L-DPPHR was oncological equivalent to PD even in the setting of selected pNET with small size. And it preserves the metabolic function without refractory cholangitis, which makes L-DPPHR an appealing alternative to PD for the sake of long-term quality of life.

The present study also has several limitations. Firstly, the retrospective nature and small sample size of a single center may invalidate the analysis. And comparative studies of L-PD and L-DPPHR with prospective protocols are awaited. Secondly, the optimal dosage and timing of ICG use needed to be further investigated, and more quantitative indicators should be introduced to evaluate the outcomes. Thirdly, long-term follow-up should be continued to evaluate the incidence of biliary stricture and quality of life as well.

## Conclusion

L-DPPHR was feasible and safe in selected patients, and real-time ICG imaging with proper dose and timing may greatly facilitate the identification and the prevention of biliary injury. And it was oncologically equivalent to PD with preservation of metabolic function without refractory cholangitis.
